# Case Report: Osteoclastic giant cell-rich cervical squamous cell carcinoma—the first reported case of a clinically silent early-detected keratinizing subtype with a detailed literature comparison

**DOI:** 10.3389/pore.2025.1612076

**Published:** 2025-05-26

**Authors:** Lukasz Fulawka, Beata Dawiec, Wojciech Homola, Agnieszka Halon

**Affiliations:** ^1^ Department of Clinical and Experimental Pathology, Wroclaw Medical University, Wroclaw, Poland; ^2^ Molecular Pathology Centre Cellgen, Wroclaw, Poland; ^3^ Gynaecology and Obstetrics Centre FemiMea, Bielany Wroclawskie, Poland

**Keywords:** cervical cancer, HPV, cervical squamous cell carcinoma, osteoclast-like giant cells, osteoclastic giant cell-rich squamous cell carcinoma

## Abstract

**Introduction:**

We report the first case of an asymptomatic woman with osteoclast-like giant cell-rich cervical squamous cell carcinoma (OGC-rich cervical SCC), where the detection of cancer was made possible only by routine cytological screening. The presence of OGCs in cervical SCCs is an extremely rare phenomenon, with only 8 cases reported to date.

**Case description:**

Two consecutive liquid-based cytology revealed high-grade squamous intraepithelial lesion (HSIL). Molecular testing detected HPV 18. Colposcopic findings strongly supported the clinical diagnosis of HSIL/suspicious for invasion. Histopathological examination of biopsy samples revealed typical keratinizing-type cervical SCC morphology. The patient subsequently underwent LEEP (loop electrosurgical excision procedure). Microscopic examination of resection specimen confirmed the previous diagnosis. Moreover, groups of large multinucleated cells were observed at the periphery of some invasive nests. Most of them presented the morphology of osteoclasts, whereas some giant cells were similar to Langhans cells. All the giant cells were positive for vimentin and CD68, negative for pancytokeratin. Owing to positive margins following the LEEP procedure, the patient underwent hysterectomy via the Wertheim technique. No adjuvant treatment was applied, and after the 9-month follow-up, the patient was alive with no recurrence.

**Conclusion:**

Detailed literature review revealed that our case is the first case of keratinizing-subtype cervical OGC-rich SCC. Moreover, it is the youngest (33 yo.) patient with a significantly smaller diameter than previously reported cases. Unfortunately, owing to the small number of reported cases, the analysis did not allow us to draw conclusions about the potential prognostic or predictive value of OGC-rich morphology.

## Introduction

Cervical carcinoma is the most common malignancy of the female genital tract and the fourth most common type of cancer in women, with an incidence of 14.1/100 thousand females. It is also the third leading cause of death due to cancer in women, with a mortality rate of 7.1/100 thousand females [1].

Histologically, 80%–90% of cervical cancers are squamous cell carcinomas (SCCs) [2]. The majority of cervical SCC cases (>90–95%) are HPV-associated [2]. HPV status is crucial from a clinical perspective because it is associated with a better prognosis and thus helps to prevent more aggressive therapy in patients who do not need it. For this reason, HPV status has been incorporated into the histological World Health Organization (WHO) classification of cervical SCC and adenocarcinoma [2]. HPV-associated neoplastic transformation is a well-known process leading to invasive SCC via precancerous lesions, namely, low-grade squamous intraepithelial lesion (LSIL), followed by high-grade squamous intraepithelial lesion (HSIL). It is widely accepted that 14 types of HPV have carcinogenic potential and are so-called high-risk HPV (HR HPV) types. Among HR HPV-associated lesions, HPV 16- and HPV 18-positive lesions carry the greatest risk of progression to malignancy [2].

Several histologic variants of cervical SCC have been described: nonkeratinizing, keratinizing, basaloid, papillary, warty/condylomatous, verrucous, squamotransitional, lymphoepithelial-like and spindled/sarcomatoid [2–5]. A wide variety of grading systems for cervical SCC have been used, but none of them have been universally accepted or widely adopted in clinical practice [6, 7].

The Pap smear screening strategy for cervical cancer has been widely used for several decades. In Poland, it remained the sole primary test in state-funded population screening programs until early 2025. Currently, the primary test in population-based screening is transitioning to HPV detection by PCR, with a transitional period during which both methods are used concurrently. The screening targets women aged 25–64, with Pap smears performed every 3 years and HPV testing every 5 years. Simultaneously, opportunistic screening — using both methods at shorter intervals — remains widespread in Poland. The presence of osteoclast-like giant cells (OGCs) has been reported in cancers at various anatomical locations, e.g., the skin, breast and pancreas [8]. Squamous cell carcinoma with OGCs is recognized as a histologic type of cutaneous SCC by the World Health Organization [9]. In the current WHO pancreatic tumor classification, undifferentiated carcinoma with osteoclast-like giant cells is one of the special histologic types of ductal adenocarcinoma [10]. Breast carcinoma with OGCs is considered a special morphologic pattern according to the 2019 WHO breast tumor classification and is most often reported in invasive carcinomas of no special type (ICs NST) [11, 12].

To the best of our knowledge, only eight cases of SCCs with OGCs of the uterine cervix have been reported to date (7 cases in full-text publications and 1 in a conference abstract) [8, 13–18]. Owing to its rarity, this type has not been recognized as a stand-alone histologic subtype or, at least, a special morphologic pattern recognized by the WHO [2]. This morphological phenomenon has not been mentioned in some generally respected books on gynecological pathology [4, 5]. However, in Blaustein’s Pathology of the Female Genital Tract it is mentioned that “ocasionally a foreign body giant cell reaction is observed” at the periphery of tumor nests in G1 tumors [19]. The Pathologyoutlines.com web service mentions OGCs as occasional phenomena in spindled/sarcomatoid variants of cervical SCC [3].

## Case description

We report a case of a 33-year-old white woman with a medical history of one pregnancy and cesarean section. No comorbidities were reported in the patient’s medical history and her family history was negative for cancer. The patient was asymptomatic at the time of the visit (May 2024). The patient reported that she was vaccinated with a quadrivalent vaccine, Gardasil (against HPV types 6, 11, 16, and 18), a few years ago. The medical record indicates that the results of the previous annual cervical cytology screening were reported as NILM (negative for intraepithelial lesion or malignancy). The only exception was an HPV 56 infection detected in 2011, which was followed by colposcopy and histopathologic examination, revealing chronic inflammation.

The patient came to the clinic for colposcopic evaluation due to HSIL (high-grade squamous intraepithelial lesion) results in two consecutive liquid-based cytology (LBC) in March and April 2024. Both LBC examinations were conducted with BD SurePath (Becton Dickinson, United States) via the automatic preparation system BD Totalys SlidePrep (Becton Dickinson, United States). The second LBC was used as a co-test, followed by the detection of 14 high-risk HPV types and genotyping via qPCR molecular testing with an Anyplex II HPV HR Detection kit (Seegene, South Korea), which was conducted using CFX96 Dx System (Bio-Rad, United States). Data analysis was performed using Seegene Viewer software (Seegene, South Korea). Nucleic acid isolation was performed with an Invisorb Spin Universal Kit (Invitek Molecular, Germany) according to the manufacturer’s procedure. The type of HPV 18 was detected.

**FIGURE 1:**
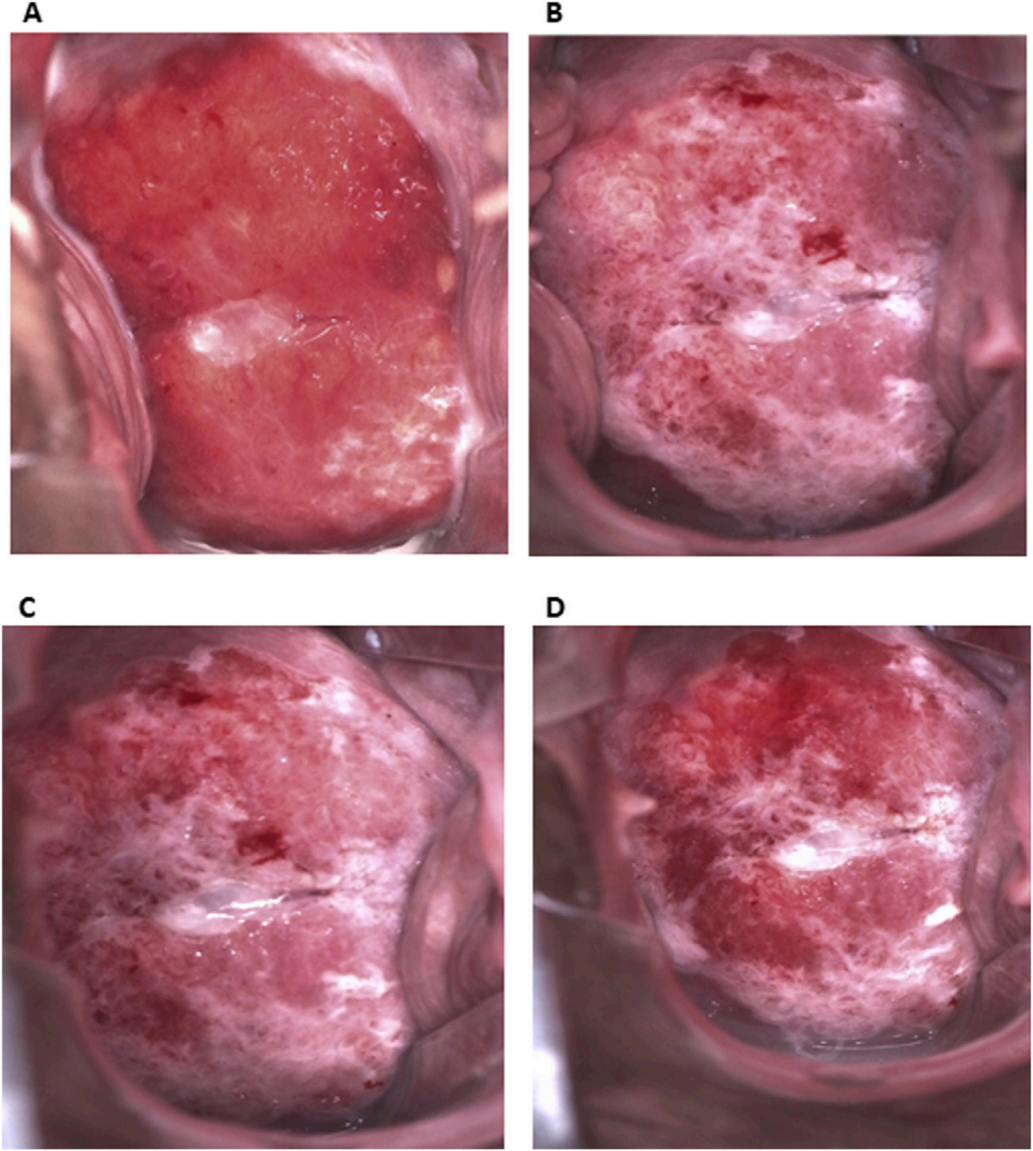
Colposcopic appearance of the lesion. **(A)** Before acid solution treatment. Extensive lesion on upper and lower lip of the cervix with abnormal vessels. **(B–D)** After acid solution treatment (B. 30 s, C. 1 min, D. 3 min). Dense acetowhite epithelium, mostly on lower lip, and also at 11 and 3 o’clock. The ridge sign at 8 o’clock, coarse punctation at upper and right lower quadrant and an irregular surface. Slight pinpoint bleeding that may indicate presence of fragile vessels.

Colposcopy with biopsy for histopathological examination was conducted using Eva Pro digital colposcope. This examination revealed major abnormal colposcopic findings: rapid appearance of dense acetowhite epithelium, abnormal vessels, ridge sign and coarse punctation. The lesion was present on the upper and lower lips of the cervix and had an irregular surface with fragile vessels, highly supporting the clinical diagnosis of HSIL/suspicious for invasion (Figure 1A). After acid solution treatment, a rapid appearance of dense acetowhite epithelium was observed, mostly on the lower lip, and also at 11 and 3 o’clock (Figures 1B–D). The inner border sign was present on the lower lip. The ridge sign at 8 o’clock, coarse punctation at the upper and right lower quadrants of the cervix and an irregular surface were the major abnormal colposcopic findings. Slight pinpoint bleeding that may indicate the presence of fragile vessels was visible. Targeted biopsy was taken from 3, 5, 8, 10, and 11 o’clock. Endocervical curettage was performed.

Histopathological examination revealed the full thickness of highly atypical squamous epithelial cells, with deep involvement of the endocervical glands, making the diagnosis of high-grade squamous intraepithelial lesion/carcinoma *in situ* (HSIL/CIS). However, the biopsy was too shallow, and the specimen was too fragmented to be examined for possible tumor invasion.

One month after colposcopy, the patient underwent LEEP/LLETZ (loop electrosurgical excision procedure/large loop excision of the transformation zone), followed by histopathological examination. The patient was informed that, owing to the extent of the transformation zone, there is a risk of nonradical resection of the lesion. The procedure was performed under local anaesthesia. Each specimen was taken from the upper and lower lips separately. There were no complications. On gross examination, two oriented fragments of the upper and lower lips were detected, measuring 2.6 × 1.6 × 0.8 cm and 2.5 × 1.4 × 0.8 cm, respectively. Barely visible slight discolouration of the mucosa surrounding the external os area on the lower lip was observed. The specimens were painted, and multiple sections were submitted according to the grossing procedure.

**FIGURE 2:**
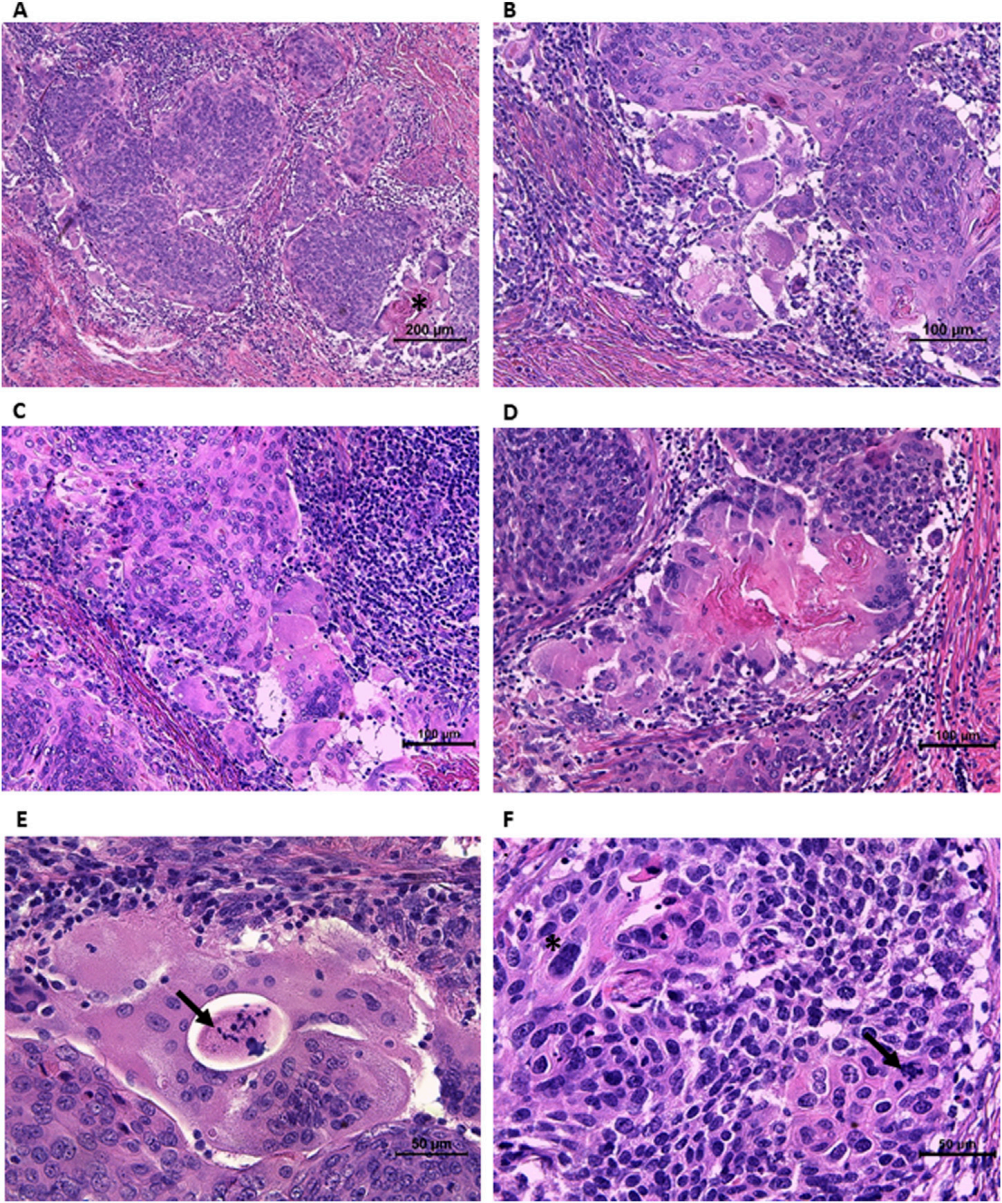
Morphological features of invasive component. **(A)** Nests of large cells with abundant slightly eosinophilic cytoplasm, maturating towards the edges of the nests, surrounded by lymphocytic infiltration. Keratin pearls (asterisk) surrounded by giant cells with peripherally located nuclei, resembling Langhans giant cells. **(B)** Mature cells at the periphery of the nest with distinct intercellular borders. The group of multinucleated giant cells of both osteoclast-like and Langhans-like morphology. **(C)** The group of osteoclast-like giant cells at the periphery of the nest. The stroma infiltrated with lymphocytes. **(D)** Higher magnification of keratin pearl surrounded by Langhans-like and osteoclast-like giant cells. **(E)** Large multinucleated cell with necrotic area and cellular debris in the middle (arrow) (probably the area outside the cell surrounded by its cytoplasm). **(F)** The area of highly atypical cells, some of them pleomorphic with a few large bizarre nuclei (asterisk) and atypical mitoses (arrow).

On microscopic examination, the invasive tumor component was easily recognizable and surrounded by HSIL at the edges. The depth of invasion was 4.5 mm, “measured from the deepest focus of tumor invasion to the base of the nearest dysplastic crypt,” according to recommendations [6]. The highest horizontal dimension of the invasive component was 10.5 mm. Nests of large cells with abundant eosinophilic cytoplasm, atypical nuclei, distinct intercellular borders, and maturation towards the edges of the nests were visible (Figures 2A,B). Keratin pearls, some of which were large, were observed throughout the tumor. Moreover, areas of highly atypical cells with poor cytoplasm and crowded nuclei were detected, some of which were pleomorphic with a few large bizarre nuclei (Figure 2F). Mitotically active regions were present, including atypical mitoses (Figure 2F).

On a closer look, groups of large multinucleated cells were observed at the periphery of some nests. They contained slightly eosinophilic cytoplasm and from a few to a few dozen nuclei. Most of them had nuclei scattered throughout the cytoplasm, making them similar to osteoclasts or foreign body giant cells (Figures 2B–D). Moreover, in some giant cells, the nuclei were located peripherally and arranged in a horseshoe-shaped pattern resembling Langhans giant cells (Figures 2A,B,D). Giant cells were located at the edges of the tumor nests or formed aggregates. Some groups of both types of multinucleated giant cells surrounded large keratin pearls (Figures 2A,D). The lymphocytic infiltration in the stroma surrounding the tumor nests was clearly visible, with only scattered intratumoral lymphocytes. Vascular and perineural invasion were not detected. However, a second-look examination revealed one area suspicious for lymphovascular invasion (necrotic tissue within an endothelium-lined space).

**FIGURE 3:**
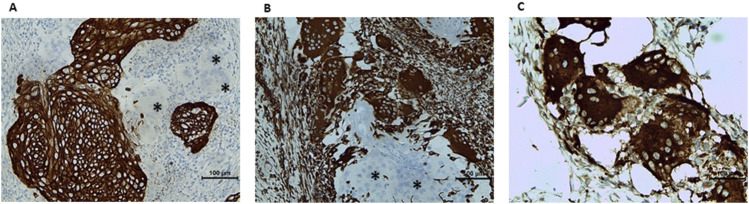
Immunohistochemical characterization of the lesion. **(A)** Pancytokeratin: positive in tumor cells, negative in multinucleated cells (asterisk). **(B)** Vimentin: negative in tumor cells (asterisk), positive in multinucleated cells. **(C)** CD68-positive osteoclast-like giant cells.

Immunohistochemistry was performed automatically on a Benchmark Ultra (Roche Diagnostics, Switzerland) with the following antibodies: pancytokeratin (clone AE1/AE3/PCK26, Roche Diagnostics, Switzerland), vimentin (clone V9, Roche Diagnostics, Switzerland) and CD68 (clone KP-1, Roche Diagnostics, Switzerland). The tumor cells were positive for pancytokeratin, negative for vimentin and CD68 (Figures 3 A–C). Whereas OGCs were positive for vimentin and CD68, negative for pancytokeratin. A final diagnosis of osteoclastic giant cell-rich squamous cell carcinoma, HPV-associated, G2, pT1a2 NX MX with positive surgical margins was made. Given that a universally accepted grading system of cervical SCC does not exist, the grade assessment of this tumor is described in the Discussion section.

Owing to positive margins following the LEEP procedure and the patient’s oncological anxiety, the patient was admitted to the gynaecological ward one and a half months later (July 2024) to undergo a more radical excision. Preoperative TV USG (transvaginal ultrasound) revealed a hypoechogenic lesion on the left side of the cervix, with dimensions of 21 × 12 × 25 mm, involving the cervical canal but not reaching the uterine body. The lesion demonstrated low vascularity. The parametrium was free of lesions, and the iliac vessel area showed no enlarged lymph nodes. The patient was deemed eligible for planned surgery, including microstaging of the lymph nodes. In the first stage of surgery, an intraoperative biopsy of the sentinel lymph nodes was performed via laparoscopy. Indocyanine green (ICG) was injected into the cervix at the 3 and 9 o’clock positions to facilitate lymphatic mapping. Histopathological examination of intraoperative frozen sections revealed no cancer cells. In the subsequent stage, a radical hysterectomy using the Wertheim technique was performed, including total resection of the uterus with the parametrium, bilateral uterosacral ligaments, and pelvic lymphadenectomy via laparotomy. The patient had no symptoms after the procedure. Routine laboratory tests revealed no abnormalities. The postoperative histopathological diagnosis was squamous cell carcinoma, HPV-associated, G2, pT1a1 N0 MX. The depth of invasion was 2.8 mm, and the highest horizontal dimension of the invasive component was 3.2 mm. Lymphovascular and perineural invasion were not detected. In total, 21 pelvic lymph nodes were resected, none of which contained tumor metastases. A focus of endometriosis was found in the right parametrium. After a full histopathological report, it was decided that adjuvant treatment was not necessary.

## Discussion and conclusion

To date, 8 cases of OGC-rich squamous cell carcinoma of the uterine cervix have been reported (7 cases in full-text publications and 1 in a conference abstract) [8, 13–18]. All the authors of these previous papers are consistent with the view of the immune cell-derived nature of OGCs. All of them reported that OGCs were negative for epidermal markers, most notably pancytokeratin, and positive for mesenchymal (vimentin) and monocyte lineage (CD68) markers. The nonneoplastic nature of OGCs was also confirmed by the nonatypical morphology of cell nuclei in all of the reports [8, 13–18].

The etiopathology of OGC-rich SCC of the uterine cervix is another interesting factor. Only 3 cases, incl. present, have had HPV etiology confirmed (Table 1). However, in more than half of the cases (5/9 cases), the HPV status has not been reported. Therefore, 75% of reported cases of known HPV status (3/4 cases) were HPV-positive. In general, more than 90% of SCCs of the uterine cervix are HPV-associated [2]. Interestingly, two different types of HR HPV were detected in the medical history of the present case. The molecular testing for HR HPV conducted in 2024 as a co-test detected HPV 18. However, according to medical interview, HPV 56 infection was detected in 2011. There are two solutions to this phenomenon.

**TABLE 1:** The clinico-pathologic characteristics of reported cases of OGCs-rich cervical SCC.

Case no.	First author, year	Country	Age (years)	Tumor diameter (mm)	Growth pattern	Stage (FIGO)	HPV PCR	Histological type	Treatment	Status	Follow- up period	IHC staining of OGCs	Ref.
1	Pang, 1998	Taiwan	65	60	Exophytic	IB2	Not reported	Sarcomatoid	SP, RT and CT	DOD	7 weeks	CD68	[15]
2	Pang, 1998	Taiwan	61	50	Exophytic	IB2	Not reported	Sarcomatoid	SP, RT and CT	DOD	14 months	CD68	[15]
3	Singh et al., 2012	India	60	45	Infiltrating	IB2	Negative	Non-keratinizing	RT and CT	ACR	6 months	CD68	[14]
4	Yu et al., 2014	China	84	50	Exophytic	IB2	Not reported	Non-keratinizing	RT	DOD	8 months	CD68, vimentin	[17]
5	Alemán-Meza et al., 2014	Mexico	49	27	Exophytic	IB1	Not reported	Non-keratinizing	SP	ACR	7 months	CD68, vimentin	[16]
6	Dejima et al., 2020	Japan	49	25	Infiltrating	IB1	16	Non-keratinizing	SP, RT and CT	ACR	22 months	CD68, CD204	[13]
7	Tziakou et al., 2020	Greece	79	no data av^a^	Exophytic	no data av^a^	no data av^a^	Non-keratinizing	RT	DOD	8 months	CD68, vimentin	[18]
8	Castillo et al., 2024	Spain	38	35	Exophytic	IB1	34	Non-keratinizing	SP	ACR	24 months	CD68, vimentin	[8]
9	Present case, 2024	Poland	33	10.5^b^	Superficial	IB1	18 (56)^c^	Keratinizing	no adj. Treatment	ACR	9 months^d^	CD68, vimentin	-

^a^ No data available - conference abstract.

^b^ Microscopic measurement.

^c^ HPV 18 was detected simultaneously with HSIL in co-test (HPV 56 was detected in the past according to medical interview) - see the Discussion section.

^d^ since HSIL result in LBC; IHC, immunohistochemistry.

RT, radiotherapy; CT, chemotherapy; SP, surgical procedure; DOD, dead of disease; ACR, alive with complete regression.

First, in our experience, discrepancies in HPV genotyping results between different test kits or laboratories—even among reputable facilities using validated methods—can be substantial (unpublished results from an interlaboratory comparison involving 350 women). Unfortunately, we lack detailed information about the 2011 test, including the qPCR kit used (assuming that qPCR was used, which was already predominant at that time), nucleic acid isolation kit, type of preservative solution and the credibility of laboratory conducting the test. Second, a biological explanation may be considered. The 13-year interval between the two tests raises the possibility of sequential infections. It is theoretically plausible that the patient first cleared the HPV 56 infection and later acquired a new HPV 18 infection. Only the latter may have led to viral genome integration and oncogenesis. However, it generally takes 15–30 years for persistent HR HPV infection to progress to invasive cervical carcinoma [2, 20]. Of note, the woman in our case was vaccinated a few years before the detection of HSIL and cancer. As reported in the literature, vaccination does not eliminate established HPV infections or prevent progression of pre-existing HPV-related lesions, but it may reduce the risk of acquiring new infections [21]. To the best of our knowledge, the present case is the first reported OGC-rich cervical SCC of the keratinizing subtype. Two-thirds (6/9 patients) had nonkeratinizing morphology, and approximately 20% (2/9 patients) had sarcomatoid morphology (Table 1). This is not surprising because, in general, the nonkeratinizing pattern is the most frequently identified subtype of cervical SCC [2, 4, 5]. The other authors observed multinucleated giant cells interspersed between tumor cells [8, 16, 17]. In contrast, the giant cells in the present case were located peripherally to the tumor nests or formed aggregates. We did not detect individual giant cells scattered over the tumor area. None of the authors have described the peripherally located nuclei of multinucleated cells, resembling Langhans giant cells. However, detailed analysis of the images presented in these papers revealed cells of this morphology, incl. The horseshoe-shaped pattern, resembling Langhans giant cells, which for some unknown reason, was not reported by any of the authors [8,16,17]. Lymphocytic infiltration was revealed in all published cases, although we observed only few intratumoral lymphocytes, in contrast to the other authors’ observations [8, 16, 17].

Grading of cervical SCC is a sensitive subject since a wide variety of grading systems have been used, and none of them have been universally accepted and adopted in clinical practice [6, 7]. This is because none of them has been proven to be reproducible or prognostically meaningful [3, 6, 7]. Interestingly, however, this does not prevent the general consensus that three classical grades are used: G1 (well differentiated), G2 (moderately differentiated) and G3 (poorly differentiated) [6, 19]. Some experts have recommended a modified historical Broder’s system, which is based on the degree of keratinization, cytological atypia, and mitotic activity [7, 19]. Most of our case consists of large cells with abundant cytoplasm, forming distinct keratin pearls. This would speak for G1. However, areas of high-grade cells with poor cytoplasm and crowded nuclei, resembling HSIL, were detected deep in the tumor. Moreover, areas of multinucleated pleomorphic cells containing large bizarre nuclei and high mitotic activity, including pathological mitoses, were detected. In view of the above, we classified the case arbitrarily as G2 (moderately differentiated).

Previous cases of OGC-rich squamous cell carcinoma of the uterine cervix were reported in middle- and old-aged patients, with only one case in a person younger than 40 years [8] (Table 1). To our knowledge, the present patient is the youngest person (33 yo.) with this type of tumor reported. What is interesting, both present and the other one below 40 yo. Came from Europe, making them two out of three cases reported in the Old Continent. However, given the small number of reported cases, this is most likely the work of chance.

All the previously reported cases of OGC-rich cervical SCC were symptomatic. Irregular vaginal bleeding was the most commonly reported clinical manifestation, followed by weight loss, whereas other symptoms were reported in single cases: abdominal bloating and coitorrhagia (bleeding after sexual intercourse) [8, 13–18]. Therefore, the present case is the first case of clinically silent cervical SCC with OGCs. The early detection of invasive cancer in the present case was made possible only by routine cytological screening. These findings provide strong evidence of the effectiveness of screening for cervical cancer. Owing to the early stage of malignancy, the medical council decided that no further treatment was required after radical hysterectomy, making it the first case reported without adjuvant treatment. We may assume that the lack of clinical manifestations in the present case is consistent with the significantly smaller diameter of the lesion (10.5 mm) than in previous cases. The diameters of the previous cases were within the range of 25–60 mm.

Owing to the small number of reported cases, it is impossible to draw conclusions about the potential prognostic or predictive value of OGC-rich morphology. The other factor that makes this impossible is the fact, rightly noted in the work of Castillo et al., that the reported cases had too short follow-up periods [8]. Moreover, the reported cases have other clinicopathological features that could potentially affect patient prognosis. The author of the first 2 cases reported dead of disease [15]. In contrast, the death rate of the following cases was less than 30% (2/7 patients) (Table 1). The two first reported cases with poor outcomes had sarcomatoid component, which was appropriately noted in the review by Castillo et al. [8, 15]. The interval between the first two and the remaining reported cases was 14 years. In addition to morphology, the change in the death rate may reflect the global decrease in cervical cancer death, owing to tremendous progress in prevention and treatment. Progress in prevention is apparent in the fact that the first four cases were FIGO IB2, whereas the following four, including the present case, were FIGO IB1 (one case reported in the conference abstract lacks data [18]). Our case was classified as FIGO IB1 because the colposcopic appearance highly supported the clinical diagnosis of HSIL/suspicious for invasion (FIGO IA is limited to “invasive carcinoma that can be diagnosed only by microscopy” [22]). Furthermore, the fatal outcome in the subgroup of the later-reported nonsarcomatoid cases involved women 79 and 84 yo., whereas the other cases were younger than 50 years. These findings support the view that age may be an additional causal factor of poor prognosis in this subgroup.

In view of the facts described in this paragraph, it is therefore understandable that OGC-rich squamous cell carcinoma of the uterine cervix has not yet been recognized as a special histologic subtype either by the WHO or any other group of experts. Further studies are needed to better understand the biological behavior and long-term prognosis of this rare subtype. We hope that this work will encourage pathologists who encounter this phenomenon during routine diagnostic practice to report their cases. Given the low prevalence of this morphologic pattern, each reported case is of considerable value.

## Data Availability

The original contributions presented in the study are included in the article/supplementary material, further inquiries can be directed to the corresponding author.

## Abbreviations

ACR, alive with complete regression; CT, chemotherapy; DOD, dead of disease; FIGO, International Federation of Gynecology and Obstetrics; HPV, human papillomavirus; HR HPV, high-risk human papillomavirus; HSIL, high-grade squamous intraepithelial lesion; IC NST, invasive carcinoma of no special type (breast); IHC, immunohistochemistry; LBC, liquid-based cytology; LEEP, loop electrosurgical excision procedure; LLETZ, large loop excision of the transformation zone; LSIL, low-grade squamous intraepithelial lesion; NILM, negative for intraepithelial lesion or malignancy; OGC, osteoclast-like giant cell; qPCR, quantitative polymerase chain reaction; RT, radiotherapy; SCC, squamous cell carcinoma; SP, surgical procedure; TV USG, transvaginal ultrasound; WHO, World Health Organization.
